# The Worker and His Hour of Need

**Published:** 1924-05

**Authors:** 


### THE WORKER AND HIS HOUR OF NEED.

Amid all the entanglements of an increasingly complex state of existence, at least one simple factor remains fairly constant. The great mass of the adult population receive, week by week, day by day, or hour by hour, those tokens of exchange which we call money, whereby they and those who are near and dear to them are enabled to live. The worries and anxieties of life largely belong to those periods when either the worker or the work stops. It is the recognition of this elementary fact that has led to our various schemes of insurance against, and public assistance towards, those emergencies which inflict on the worker and his dependants these ills of worry and anxiety. Periods of unemployment descend upon industries from causes which may be economic or political, or may be local, national, or international. The work stops, yet the worker has to live. If the work goes on, the worker himself comes to a standstill, sooner or later, through accident, illness, old age or death. The wage-earners of the country want provision against all these emergencies, and their self-respect demands that they should themselves contribute substantially towards the cost of such provision.

The various schemes for meeting these contingencies have either grown up piecemeal or have not yet passed the region of promises. Those which exist are gravely incomplete. They are administered in a variety of ways. There is everywhere an absence of plan. Subsistence during unemployment has been met in some cases by insurance, in others by dole, in others by the Poor Law. National Health Insurance takes account, and that only partially, of the needs of the worker during sickness, but does not extend to the needs of his family. In both schemes of insurance, whether against sickness or unemployment, the cost falls partly on the employer, partly on the employed person, and partly on the State, with a further facile resort to the pockets of the State when difficulties arise. Old Age Pensions fall wholly upon the State; insurance against accident is made the concern of the employer; provision against the contingency of death is left for the wage-earner to make, if he thinks fit. Pensions for the mother and the widow, presumably from the State, are promised. Various agencies are at work in the administration of all these matters—the Labour Exchanges, the Approved Societies under the Health Insurance scheme, including the old Friendly Orders, the Trade Unions, the Industrial Insurance Companies.

In all this welter of administration there is inevitably overlapping and unnecessary expense. No wage-earner can safely ignore any of the emergencies to which we have referred, or, to the extent to which he does so, a confused and irrational burden falls in some haphazard way upon the community. There are plenty of enthusiasts ready to dip into the pockets of the State and to " dope " the wage-earners with this form of assistance, as though it were a panacea for the ills themselves. These dippers and dopers, if they had their way, would succeed only in making confusion worse confounded; it will be a healthy state of mind, if we ever attain to it, in which unqualified State assistance will be regarded in much the same way as Poor Law relief. On the other hand, recognition of the facts to which we have referred is leading the thinking members of the community to demand unification and extension of the existing schemes of social insurance. This demand appears to us to be soundly conceived, and we are entirely in agreement with the view set forth by a recent contributor to the *Times*, in an able and thoughtful article, in which he suggests, as a first step, that the whole subject should be examined by a Royal Commission. It seems unfortunate that the proposed Royal Commission on Health Insurance should have the issue before it narrowed to what is part only of one very considerable social problem of to-day.

